# Genome-wide identification and expression analysis of glutathione S-transferase gene family to reveal their role in cold stress response in cucumber

**DOI:** 10.3389/fgene.2022.1009883

**Published:** 2022-09-29

**Authors:** Xiaoyu Duan, Xuejing Yu, Yidan Wang, Wei Fu, Ruifang Cao, Lu Yang, Xueling Ye

**Affiliations:** ^1^ Key Laboratory of Protected Horticulture of Education Ministry and Liaoning Province, College of Horticulture, Shenyang Agricultural University, National and Local Joint Engineering Research Centre of Northern Horticultural, Facilities Design and Application Technology (Liaoning), Shenyang, Liaoning, China; ^2^ College of Life Science, Shenyang Normal University, Shenyang, Liaoning, China

**Keywords:** glutathione S-transferases, cucumber, cold stress, glutathione metabolism, transcriptome

## Abstract

The plant glutathione S-transferases (GSTs) are versatile proteins encoded by several genes and play vital roles in responding to various physiological processes. Members of plant GSTs have been identified in several species, but few studies on cucumber (*Cucumis sativus* L.) have been reported. In this study, we identified 46 GST genes, which were divided into 11 classes. Chromosomal location and genome mapping revealed that cucumber GSTs (CsGSTs) were unevenly distributed in seven chromosomes, and the syntenic regions differed in each chromosome. The conserved motifs and gene structure of CsGSTs were analyzed using MEME and GSDS 2.0 online tools, respectively. Transcriptome and RT-qPCR analysis revealed that most CsGST members responded to cold stress. Gene Ontology (GO) and Kyoto Encyclopedia of Genes and Genomes (KEGG) pathway enrichment analyses for differentially expressed CsGSTs under cold stress revealed that these genes responded to cold stress probably through “glutathione metabolism.” Finally, we screened seven candidates that may be involved in cold stress using Venn analysis, and their promoters were analyzed using PlantCARE and New PLACE tools to predict the factors regulating these genes. Antioxidant enzyme activities were increased under cold stress conditions, which conferred tolerance against cold stress. Our study illustrates the characteristics and functions of CsGST genes, especially in responding to cold stress in cucumber.

## Introduction

Glutathione S-transferases (GSTs; EC 2.5.1.18) are phase II metabolic isozymes ubiquitous in fungi, animals, and plants (Dixon et al., 2002). The first description of GSTs in plants dates back to 1970, where a study demonstrated that GSTs isolated from maize were involved in herbicide metabolism through catalyzing the conjugation of synthetic compounds with glutathione [GSH; γ-Glu–Cys–Gly; (Frear and Swanson, 1970)].

Plant GST proteins are globular dimers, usually containing two distinct N- and C-terminal domains. The N-terminal domain consists of a structural element of β-strands and α-helices arranged in a thioredoxin-like fold, in which a GSH binding site (G-site) with high substrate specificity exists. The C-terminal domain consists entirely of α-helices, in which an electrophile substrate binding site (H-site) with hydrophobic characteristics and broad substrate specificities exists. This site accepts various substrates and ligands (Dixon et al., 2002; Dixon and Edwards, 2010). According to previous studies on several plant GSTs, GSTs can be divided into 14 classes, including phi, zeta, tau, theta, lambda, Ure2p, EF1Bγ, hemerythrin, iota, glutathionyl-hydroquinone reductases (GHRs), dehydroascorbate reductase (DHAR), tetrachlorohydroquinone dehalogenase (TCHQD), metaxin, and microsomal prostaglandin E synthase type 2 (mPGES-2) (Lallement et al., 2014; Nianiou-Obeidat et al., 2017; Wei et al., 2019; Hasan et al., 2021). Among these classes, tau, phi, DHAR, and lambda are plant-special GSTs. The tau and phi classes are the most abundant, probably due to their rapid evolution in plants. For example, the *A. thaliana* GST gene family includes 57 members divided into nine classes, among which the number of phi and tau members is 13 and 28, respectively (Dixon et al., 2009).

Plant GSTs are versatile enzymes involved in various biological processes such as xenobiotic detoxification, secondary metabolism, and biotic and abiotic stresses (Estévez and Hernández, 2020). For example, *TcGSTd2* and *TcGSTd3* respond to phoxim and lambda-cyhalothrin, suggesting that they play a crucial role in detoxification (Song et al., 2020). Recently, plant GSTs associated with anthocyanin accumulation have received wide attention. IbGSTF4 in sweet potato (Kou et al., 2019), MdGSTU12 in apple (Zhao et al., 2021), PcGST57 in pear (Li B.et al., 2022), PpGST1 in peach (Zhao et al., 2020), and GhGSTF12 in cotton (Shao et al., 2021) could modulate anthocyanin transport and accumulation. Furthermore, *PbrGSTs* are involved in the development of superficial scald by regulating redox balance in Chinese white pear (Wang et al., 2018). Considering biotic and abiotic stresses, the GST gene, *LrGSTU5*, has been transferred and expressed into tobacco (*Nicotiana tabacum*) during biotic stress, resulting in transgenic tobacco exhibiting enhanced resistance to *F. oxysporum* by significantly increasing antioxidant enzyme activity and decreasing superoxide anion (O_2_^−^) production (Han et al., 2016). Overexpression of *PtGSTF4* in *A. thaliana* enhances the ability to tolerate drought and NaCl stresses in transgenic *A. thaliana* lines (Yang et al., 2019). The GST gene, *JrGSTTau1*, has been regarded as a candidate for improving the chilling tolerance of plants by increasing enzyme activity, scavenging reactive oxygen species (ROS), and inducing stress-related genes (Yang et al., 2016). In addition, plant GSTs play a vital role in alleviating heavy metal toxicity, including that of Cu, Cd, Cr, and Pb (Li et al., 2018; Gao et al., 2020).

Cucumber (*Cucumis sativus* L.) is an economically important crop that is widely cultivated in tropical regions. It is chilling-sensitive and exhibits chilling injury symptoms when subjected to cold stress. Cold stress is an environmental stress that limits plant growth and development, and causes much damage at biochemical, physiological, metabolic, and molecular levels in plants (Sudesh, 2010). When plants are subjected to cold stress, the accumulation of ROS is considerably accelerated, resulting in the oxidative destruction of plant cells (Asada, 2006). However, plants can eliminate ROS to protect themselves from damage *via* non-enzyme and enzyme antioxidant defense systems (Montezano and Touyz, 2014). The non-enzyme defense system comprises ascorbate (AsA), carotenoids, tocopherols, glutathione (GSH), proline, and soluble sugars, among others (Theocharis et al., 2012; Fedotova and Dmitrieva, 2016; Surówka et al., 2020). Meanwhile, the enzyme defense system includes superoxide dismutase (SOD), catalase (CAT), ascorbate peroxidase (APX), glutathione reductase (GR), and GSTs (Gill et al., 2013; Adhikari et al., 2022). As antioxidative enzymes, GSTs play a significant role in detoxifying ROS produced within cells (Gill et al., 2013).

In this study, a genome-wide analysis of GST genes in cucumber was conducted, and 46 members were identified. Each member was analyzed further to identify their chromosomal location, phylogenetic and syntenic relationships, conserved motifs, and gene structure. Furthermore, the expression pattern of CsGST members and the functional annotation of differentially expressed CsGSTs were analyzed under cold stress conditions. Seven candidates CsGST genes involved in cold stress were screened, and their promoters were analyzed through bioinformatics approaches. Additionally, the activity of antioxidant enzymes (such as SOD, CAT, and GR) was measured under cold stress conditions. Our findings will provide novel insights into the molecular, evolutionary, and functional characteristics of CsGST genes.

## Materials and methods

### Plant materials and growth conditions

The North China-type cucumber inbred line “JinYan No. 4” was used in this study. Cucumber seedlings were cultivated in a phytotron at 28/18°C and 16 h/8 h day and night. Daylight intensity and air humidity were maintained at 10,000 Lux and 70%, respectively. When cucumber seedlings had three true leaves, they were subjected to cold stress at 15/6°C and 12 h/12 h day and night for 24 h. The cucumber leaves were sampled after treatment at 0, 3, 12, and 24 h, frozen immediately in liquid nitrogen, and stored at −80°C until further analysis.

### Identification and characterization of glutathione S-transferases genes in cucumber

Fifty-seven full-length *A. thaliana* GST protein sequences were obtained from the TAIR database (https://www.arabidopsis.org/). To identify the CsGST genes, a BLASTP search was performed in the Cucurbit Genomics Database (http://cucurbitgenomics.org/organism/2) against *A. thaliana* GST protein sequences with a cutoff *E*-value ≤ 10^−10^. Meanwhile, we used “glutathione S-transferase” as the keyword to search for related proteins in the cucumber genome (Chinese Long V3). Thereafter, we manually integrated the putative GST proteins obtained by the two methods and removed redundant proteins. The putative cucumber GST protein sequences were downloaded and submitted to the SMART and PFAM databases (http://smart.embl.de/smart/set_mode.cgi) to identify the typical protein sequence domain. Furthermore, basic protein properties [amino acid number, theoretical isoelectric point (pI), and molecular weight] were determined using the ExPAsy (https://www.expasy.org/) online tool. The subcellular location was predicted using Plant-mPLoc (http://www.csbio.sjtu.edu.cn/bioinf/plant/) and the Uniprot database (https://www.uniprot.org/), and the molecular functions of CsGST proteins were identified using the Uniprot database.

### Nomenclature and structure analysis of CsGST genes

The methodology for naming cucumber GSTs was identical to that previously proposed by Dixon et al. (2002). Specific letters with successive numbers represent members of a unique subfamily. For example, CsGSTU, CsGSTL, CsGSTF, CsGSTZ, CsGSTT, CsDHAR, CsTCHQD, CsEF1G correspond to tau, lambda, phi, zeta, theta, DHAR, TCHQD, and EF1G classes, respectively. The numbers signify the different members of the subfamily. The gene structures of the CsGST genes, showing the number and specific location of exons and introns, were visualized on the Gene Structure Display Server (GSDS; http://gsds.cbi.pku.edu.cn/).

### Chromosomal location and syntenic region analysis

The specific location of the CsGST genes on chromosomes was queried from the cucumber genome database (Chinese Long V3) and plotted using Mapchart 2.3 software (Voorrips, 2002). After downloading the cucumber and *A. thaliana* genomes, the GST genes in the cucumber genome were aligned against the *A. thaliana* genome using BLAST. The final results were extracted using MCScanX (Wang et al., 2012) to obtain the syntenic regions between cucumber and *A. thaliana*, and the extracted data were visualized using Circos software (Krzywinski et al., 2009).

### Phylogenetic tree construction and conserved motif analysis

The GST protein sequences of *A. thaliana*, rice, pumpkin, and cucumber were prepared for phylogenetic analysis. Subsequently, multiple sequences were aligned using Clustal W, and phylogenetic trees were constructed using MEGAX software (Kumar et al., 2018), employing the neighbor-joining method with 1,000 bootstrap replicates (Felsenstein, 1985; Saitou and Nei, 1987). The conserved motifs were determined using the MEME online tool with the following parameters: number of motifs = 15; remaining parameters = default (Bailey and Gribskov, 1998).

### RNA-seq analysis

The cold-treated cucumber leaves were used for constructing complementary DNA (cDNA) libraries for RNA-seq with three biological replicates (GEO accession No. GSE210703). The RNA concentration and purity were quantified using a NanoDrop ND-1000 Spectrophotometer (NanoDrop Technologies, Wilmington, DE, United States), and RNA integrity was assessed using a Bioanalyzer 2100 Instrument (Agilent Technologies, Santa Clara, CA, United States) with a RIN number > 7.0, and confirmed by electrophoresis on a denaturing agarose gel. The quantified RNA was used to construct the cDNA library and sequenced on an Illumina Novaseq™ 6000 platform (Illumina, San Diego, CA, United States) at LC-Bio Technologies Co., Ltd., (Hangzhou, China). Clean data were obtained after removing low-quality and ambiguous bases and mapped to the cucumber (Chinese Long) genome V3 database (http://cucurbitgenomics.org/organism/20) using HISAT2 v2.0.4 software. Gene expression level was represented as FPKM, and differentially expressed genes were selected based on the thresholds of |log2FC| > 1 and *p*-value < 0.05.

### Real-time quantitative PCR analysis

The cDNA was used as the template for conducting Real-time quantitative PCR (RT-qPCR) using a Roche LightCycler 96 Instrument (Roche Sequencing, Pleasanton, CA, United States). The reaction mixture (20 μl) comprised 2 µl cDNA, 1 µl of each primer, 10 µl SYBR Green SuperMix (Takara Bio Inc., Shiga, Japan), and 6 µl ddH_2_O. Primers for the related genes were designed using the NCBI online tool Primer-Blast (https://www.ncbi.nlm.nih.gov/tools/primer-blast/) and are listed in Supplementary Table S1. The RT-qPCR program was set as follows: denaturation at 95°C for 10 min, followed by 40 cycles of 95°C for 30 s, 60°C for 30 s, and 72°C for 30 s. Finally, the relative expression of target genes was calculated using the 2^−ΔΔCt^ method.

### Analysis of regulatory elements in CsGST gene promoters

The promoter region (1,500 bp upstream of ATG) of candidates was downloaded from the cucumber genome (Chinese Long V3) and analyzed using the PlantCARE (http://bioinformatics.psb.ugent.be/webtools/plantcare/html/) and New PLACE (https://www.dna.affrc.go.jp/PLACE/?action=newplace) online tools to analyze the *cis*-acting elements in CsGST gene promoters.

### Determination of antioxidant enzyme activity

Cucumber leaves (0.2 g) were ground with a 1.5-ml chilled extraction solution containing 50 mg polyvinylpyrrolidone and 1 mM EDTA (pH 7.5), after which the mixture was centrifuged at 8,000 rpm for 20 min at 4°C, and the supernatant was collected for the determination of enzyme activity. Superoxide dismutase (EC 1.15.1.1) activity was determined according to the method of Dhindsa et al. (1981); one unit of activity was defined as the amount of enzyme needed to inhibit 50% of NBT photoreduction at 560 nm. Catalase (EC 1.11.1.6) activity was determined according to Li et al. (2021); one unit of activity was defined as the amount of enzyme required to reduce absorbance by 0.01 at 240 nm. Glutathione reductase (EC 1.6.4.2) and APX (EC 1.11.1.11) activities were analyzed according to the method of Noctor et al. (2016). Glutathione reductase activity was determined by monitoring the decrease in NADPH at 340 nm for 3 min, whereas APX activity was assayed by monitoring the decrease in ascorbate at 340 nm for 3 min.

### Statistical analysis

The data were statistically analyzed using SPSS software (v. 23; SPSS Inc., Chicago, IL, United States). Analysis of variance and Duncan’s multiple comparisons were performed to determine differences between groups. Statistical significance was considered at *p* < 0.05. Data are presented as the mean ± standard error of the mean (*n* = 3). Figures were constructed using Origin software (version 2021; OriginLab, Northampton, MA, United States).

## Results

### Identification and analysis of CsGST genes

We identified 46 cucumber GST protein sequences with a typical GST domain. However, certain members contained specific domains. For example, CsMGST1 only contained a MAPEG domain (pfam01124), similar to MGST in *A. thaliana*, with which it shared 53.61% homology. Additionally, the multiple sequences of other species were aligned, and the results indicated that the MAPEG domain was highly conserved in different species (Supplementary Figure S1). CsEF1G 1 and 2 contained an EF1G domain (elongation factor 1 gamma; pfam00647) in addition to a GST-N or GST-C domain; therefore, they were classified as EF1G class, as proposed in a previous study (Jiang et al., 2010). Notably, CsGST2N1 and CsGST2N2 lacked a GST-C domain and contained two repeated GST-N domains. The basic properties and characteristics of CsGST proteins are listed in Supplementary Table S2. The data indicated that the amino acid sequences of CsGSTs vary from 145 to 476 in length, 5.05 to 9.66 in pI, and 16.49–46.34 kDa in molecular weight.

### Gene structure analysis of CsGST genes

**FIGURE 1:**
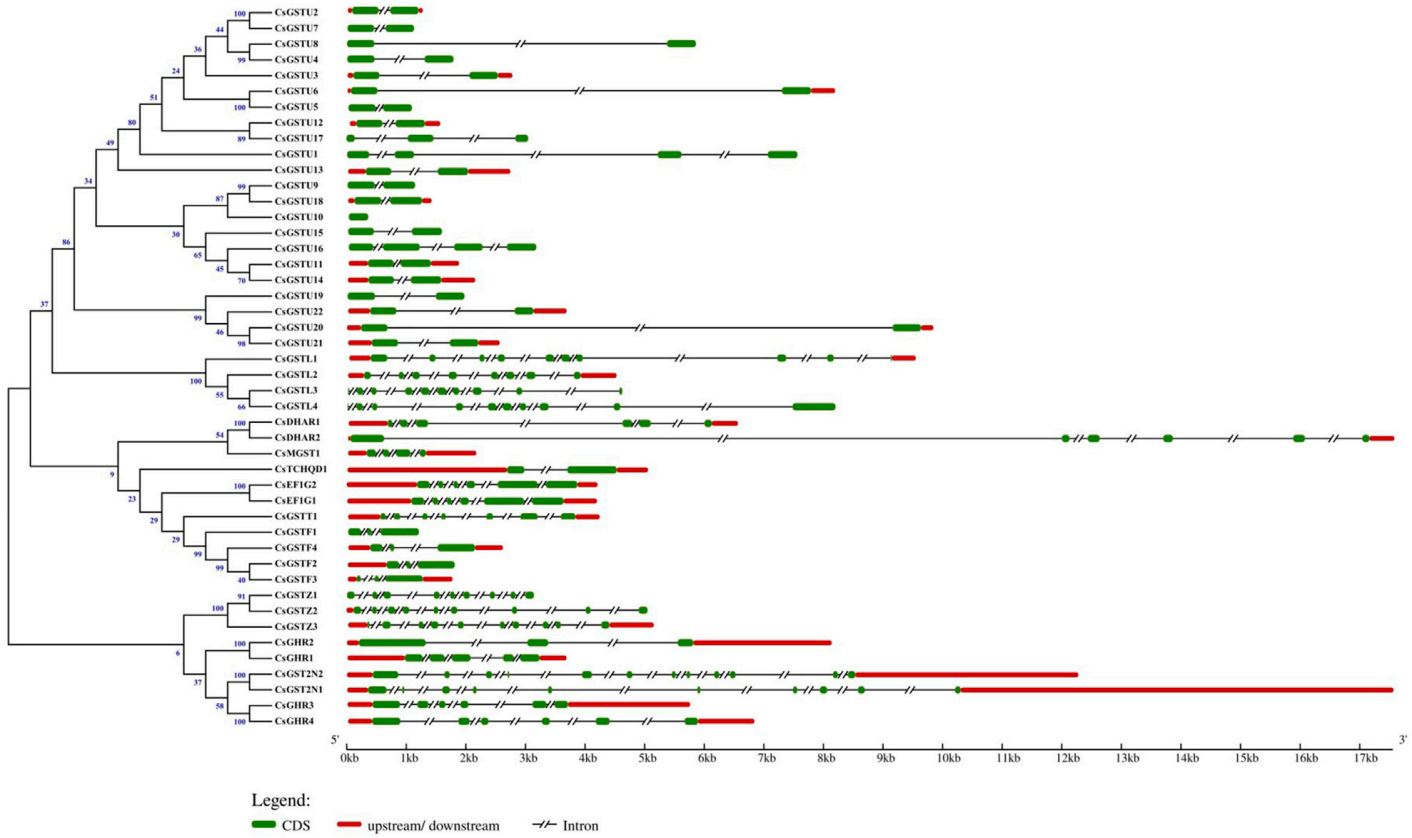
Relationship of the GST family genes in cucumber and their structure. Phylogenetic tree of the 46 identified GST genes and their structures. Exons, introns, and their upstream/downstream regions are represented by green boxes, black lines, and red boxes, respectively. The scale on the bottom shows the relative lengths of transcripts, exons, and introns in kilobase pairs (Kb).

GSDS was applied to analyze the gene structure of CsGST genes (Figure 1). The results revealed that members of the same class possess a highly conserved gene structure, whereas those of different classes exhibited diversity in exon number and position. Among the 46 CsGST members, the maximum and minimum exon numbers were observed in CsGST2N (12 exons) and CsGSTU10 (only 1 exon), respectively. In addition, exon length varied between different classes. All tau class members except CsGSTU10 showed a single intron/two exon organization, with the location of the first exon highly conserved in the N-terminal region, and all phi-class members exhibited a two intron/three exon structure. EF1G- and DHAR-class members had six exons, zeta- and lambda-class members had 8–10 exons, and CsTCHQD1 and CsMGST1 contained 2 and 4 exons, respectively.

### Chromosomal location and syntenic region analysis of CsGST genes

The specific localization of the 46 CsGST genes on chromosomes was obtained using Mapchart software (Supplementary Figure S2). The results showed that the distribution of the 46 CsGST genes on the 7 chromosomes was non-random and uneven. Both chromosomes 4 and 3 contained the largest number of CsGST genes (14 of 46; 31%), whereas chromosome 6 was devoid of CsGST genes. Chromosome 1 encoded 6 CsGST members (6 of 46, 13%), chromosomes 2 and 5 contained 5 and 4 CsGST members, respectively, and chromosome 7 contained 3 CsGST genes. CsGST genes in the same class clustered together, forming seven clusters on different chromosomes. Among them, tau-class clusters were positioned on chromosomes 3 and 4, on which members were arranged tandemly. EF1G and lambda cluster members were arranged tandemly on chromosome 3, and the zeta and phi CsGST clusters were on chromosomes 2 and 7, respectively.

**FIGURE 2:**
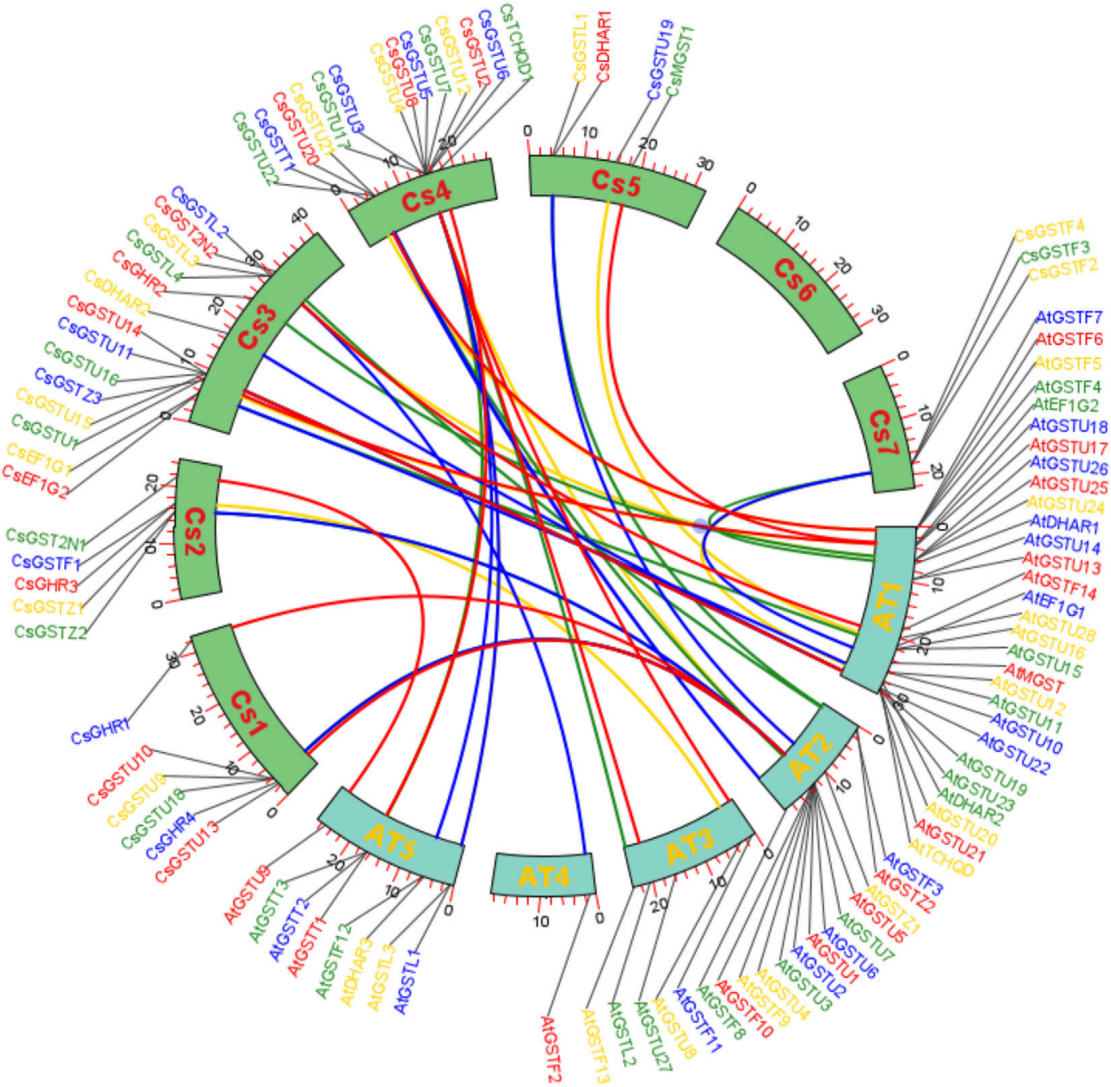
Syntenic region analysis of GST genes between cucumber and *A. thaliana*. Collinear correlations of GST gene homologs in the syntenic region in cucumber and *A. thaliana*. The different lines represent homologous proteins between cucumber and *A. thaliana.*

Syntenic regions were plotted to show the homology of GST proteins between cucumber and *A. thaliana* (Figure 2). Within the cucumber genome, chromosome 3 had the most syntenic regions (35%), followed by chromosome 4 (32%), whereas chromosome 6 had no syntenic regions. Syntenic regions on chromosomes 3 and 4 of cucumber were homologous with those on chromosomes 1 and 2 and 2 and 3 of *A. thaliana*, respectively.

### Phylogenetic analysis and conserved motifs of CsGST members

**FIGURE 3:**
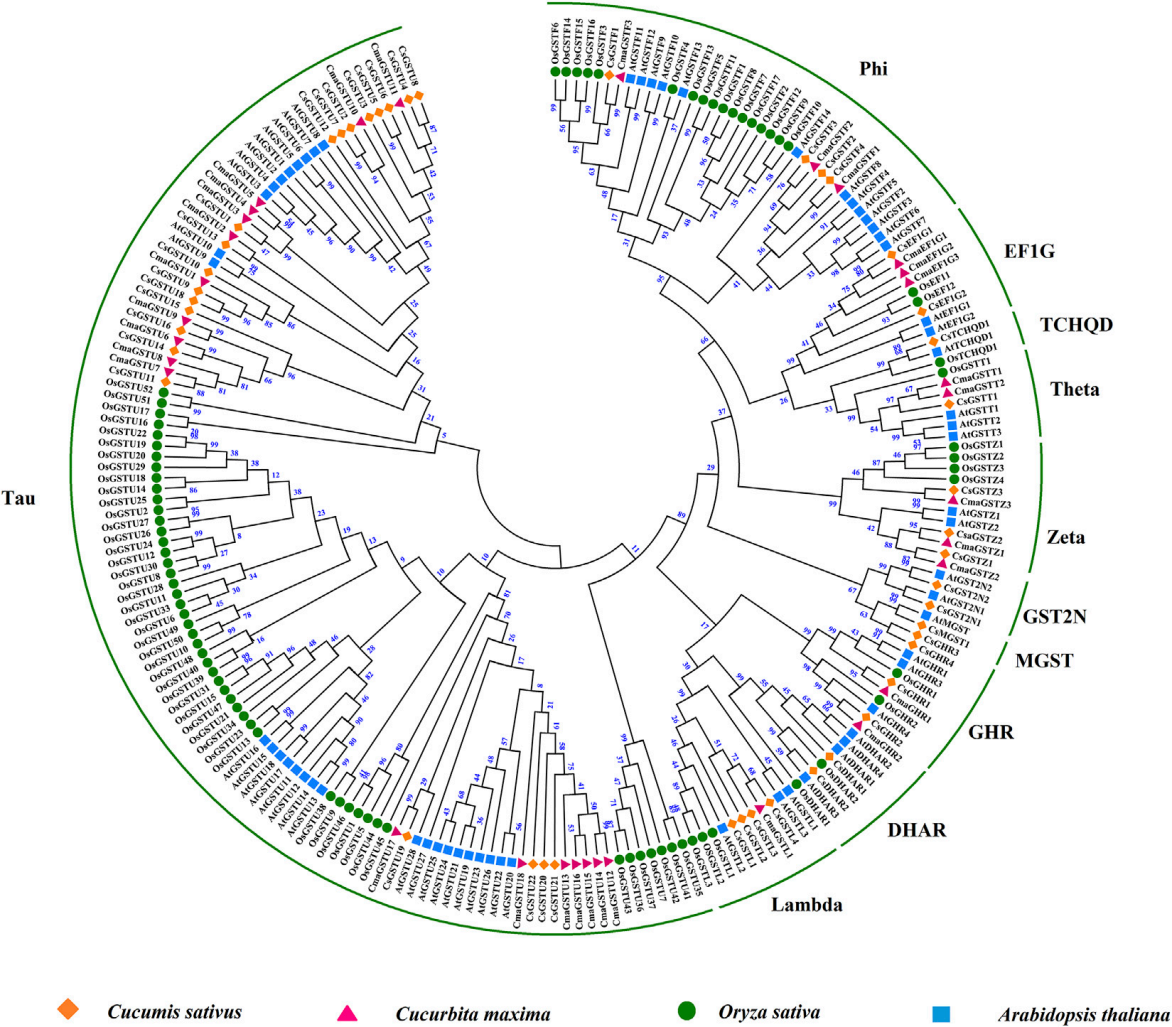
Phylogenetic analysis of GST proteins from different species. Phylogeny of the GST proteins from cucumber rice, pumpkin, and *A. thaliana*. The number on each node represents bootstrap values. The GST proteins of cucumber are divided into 11 subfamilies.

The phylogenetic tree was constructed utilizing 220 full-length protein sequences from cucumber, rice, pumpkin, and *A. thaliana*. The results showed that same classes in different species clustered together. The 46 CsGSTs in cucumber could be divided into 11 GST classes: tau, phi, lambda, zeta, theta, EF1G, DHAR, MGST, TCHQD, GHR, and GST2N (Figure 3). Among the 46 CsGSTs, tau was the largest class with 22 CsGST members, followed by phi, GHR, and lambda classes with four members each, then zeta class with three members, DHAR, GST2N, and EF1G classes with two members each, and finally theta, MGST, and TCHQD classes with only one member each.

The MEME Suite was used to analyze the conserved motifs of CsGST proteins, and 15 conserved motifs were identified (Supplementary Figures S3A,B). The longest conserved motif contained 50 amino acids and the shortest comprised 15 amino acids. Motifs in the same class members resembled each other in position and number, indicating that they may possess similar functions. For instance, EF1G class members contained motifs 1, 4, 5, 10, 12, and 15, and all lambda class members contained motifs 1, 2, 5, 8, and 11. Most CsGSTs contained motifs 1, 2, and 4, indicating that these motifs were highly conserved in CsGST genes. The tau class exhibited the largest change in motif number, varying from 3 to 17 motifs. Most tau members contained nine motifs, motifs 1–7, 9, and 10; among these, motifs 5 and 9 were unique to the tau class. Most phi members contained motifs 1, 2, 5, 13, and 14, of which motifs 13 and 14 were specifically observed in the phi class. Motifs 8 and 11 were only present in the lambda class. All CsGHR members contained motifs 2, 5, 9, and 12, with motif 12 unique to the CsGHR class. There was only a one-motif difference between CsDHAR1 and CsDHAR2; CsDHAR1 comprised motifs 1, 2, 4, 5, and 8, whereas CsDHAR2 comprised motifs 1, 2, 4, 5, and 14. CsTCHQD1 contained four motifs, namely motifs 1, 2, 5, and 14. Both CsGST2N1 and CsGST2N2 comprised three motifs; CsGST2N1 contained two motif 2s and one motif 14, whereas CsGST2N2 contained two motif 2s and one motif 1. Taken together, the existence of unique motifs in different classes may be the reason for the diverse functions of GST proteins.

### CsGST gene expression pattern analysis under cold stress

**FIGURE 4:**
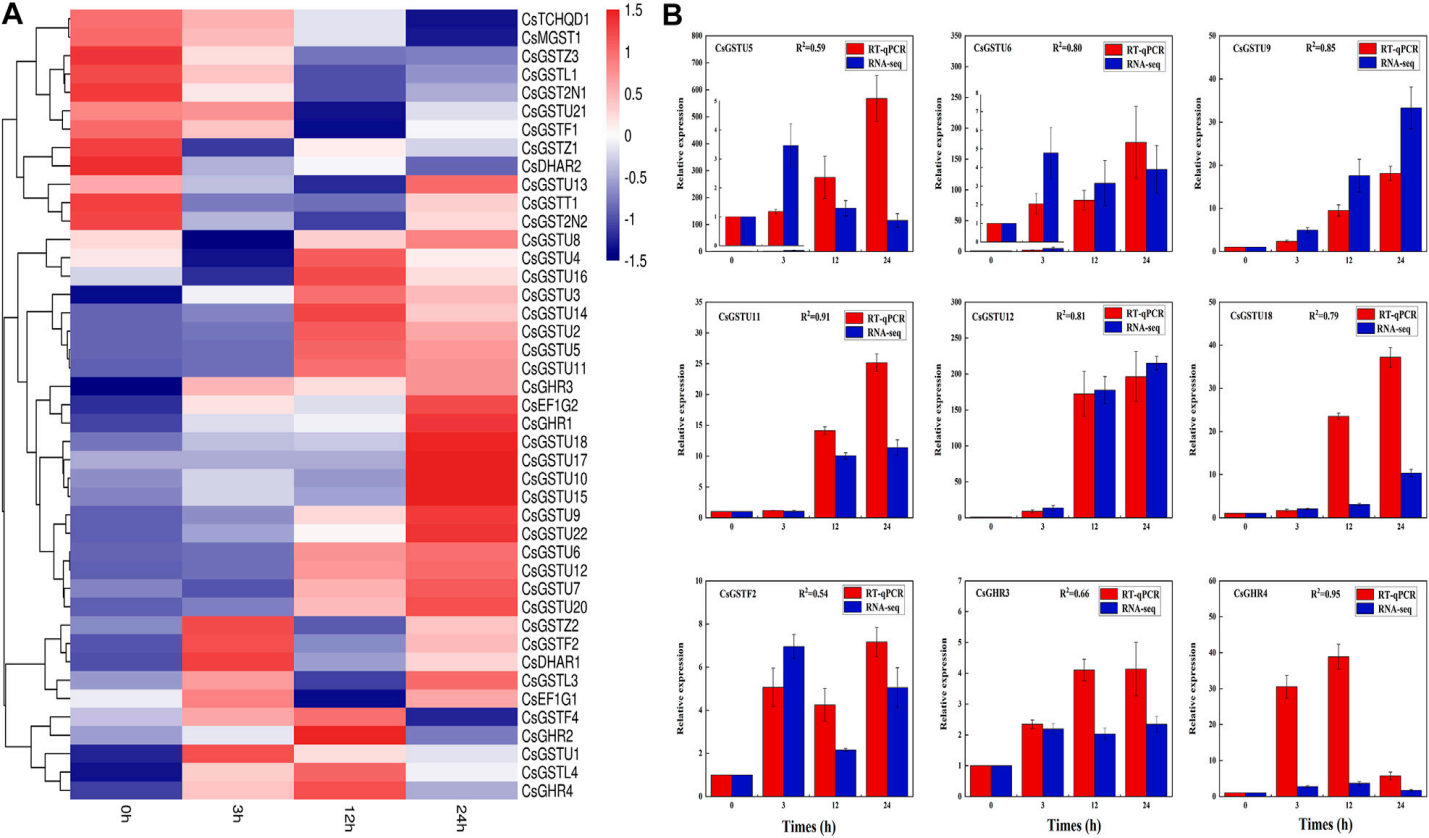
CsGST gene expression pattern and transcriptome validation in cucumber under cold stress. **(A)** CsGST genes that responded to cold stress were identified from RNA-seq data; 0 h was considered the control. Red and blue represent upregulated and downregulated genes, respectively. **(B)** Validation of transcriptome data and expression of cucumber leaf CsGSTs under cold stress. Expression analysis was performed using RT-qPCR. The expression of CsGSTs at 0 h was considered the control used for normalization. The values were analyzed from three biological replicates and represent means ± SE. R^2^ represents the correlation coefficient between RNA-seq and RT-qPCR.

According to transcriptome data, 43 CsGST genes (FPKM > 0) were detected in cucumber leaves under cold stress, and their expression patterns were divided into three distinct clades (Figure 4A). The first clade containing 12 members showed drastic downregulation as cold-treatment time progressed. The second clade containing 21 members exhibited upregulation, and the third clade containing 10 members showed an initial sharp upregulation followed by downregulation. To validate the transcriptome data, we randomly selected nine CsGST genes for expression pattern analysis using RT-qPCR (Figure 4B). The results showed that the correlation coefficients between RT-qPCR and transcriptome data varied from 0.54 to 0.95, demonstrating transcriptome data credibility.

### Functional annotation of CsGST genes under cold stress

**FIGURE 5:**
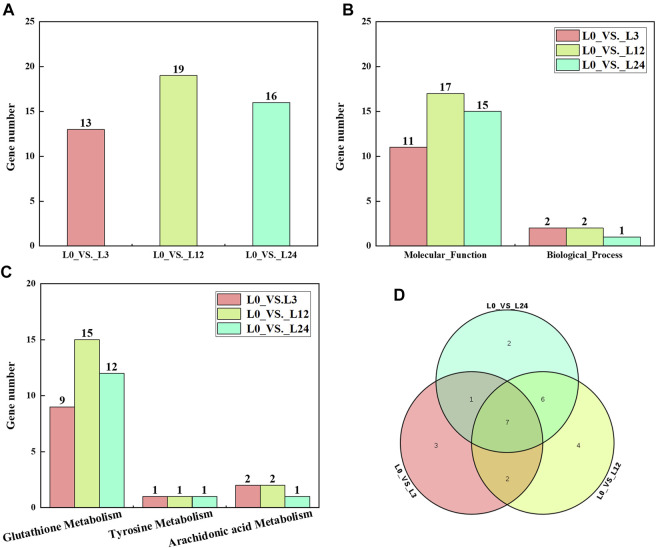
Analysis of differentially expressed CsGSTs. **(A)** The number of differentially expressed CsGSTs of different groups under cold stress. **(B)** The number of differentially expressed CsGSTs in GO annotation. **(C)** The number of differentially expressed CsGSTs in KEGG pathway enrichment analysis. **(D)** Venn diagram showing the number of common and unique differentially expressed CsGSTs among the different groups.

Compared with those at 0 h, the numbers of differentially expressed CsGST genes at 3, 12, and 24 h after cold treatment were 13, 19, and 16, respectively (Figure 5A). To further characterize their biological role, GO annotation and KEGG pathway enrichment analysis were conducted. The GO annotation results indicated that most differentially expressed CsGST genes were assigned to the “protein binding” GO term belonging to the biological process category, and few CsGST genes were assigned to the “cell redox homeostasis” GO term belonging to the molecular function category (Figure 5B; Supplementary Table S3). Furthermore, KEGG pathway enrichment analysis results showed that tau-class CsGST genes were enriched in the “glutathione metabolism” pathway, *CsGHR3* and *CsGHR4* genes were enriched in “arachidonic acid metabolism,” and *CsGSTZ*1 was enriched in “tyrosine metabolism” (Figure 5C; Supplementary Table S4). The Venn diagram showed that seven genes (*CsGSTU5*, *CsGSTU6*, *CsGSTU9*, *CsGSTU12*, *CsGSTU18*, *CsGHR1*, and *CsGHR3*) were differentially expressed during the entire cold stress period (Figure 5D). These results suggest that the seven GST genes respond to cold stress through the “glutathione metabolism” pathway in cucumber seedlings.

### Promoter analysis of putative candidate CsGST genes

**FIGURE 6:**
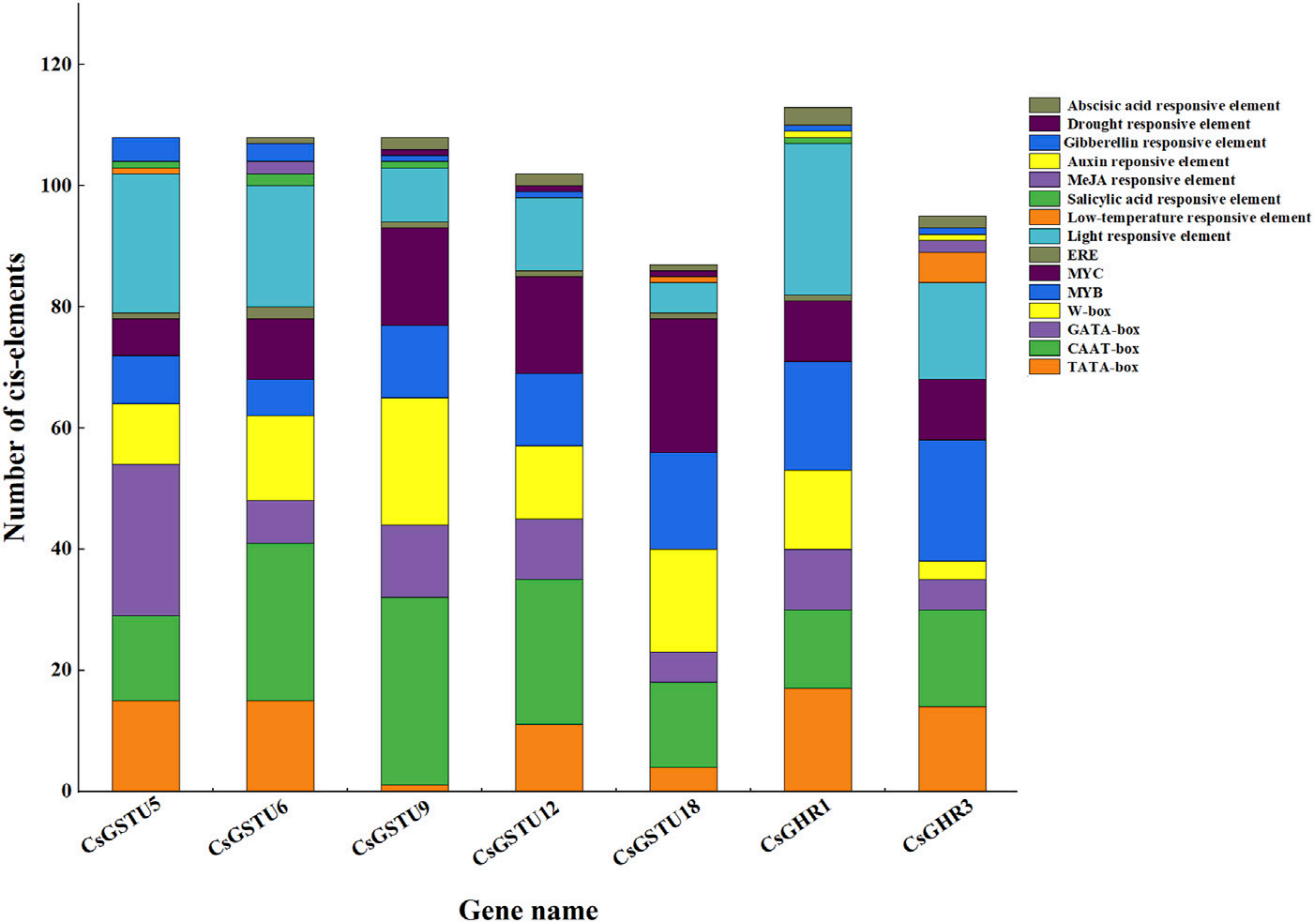
Putative cis-element analysis in promoters of the seven CsGSTs candidates. The diverse *cis*-elements are shown using different colors, and the number of different *cis*-elements is represented by rectangle height.

The promoter regions of the seven putative candidate CsGST genes were analyzed using PlantCARE and New PLACE online tools to identify the presence of *cis*-acting elements (Figure 6). Through further integration and statistics, we found that apart from the core elements (TATA, CAAT, and GATA box), certain *cis*-elements were involved in light, phytohormone signal, and stress responses. For instance, the phytohormone signal-responsive elements consisted of salicylic and abscisic acids and gibberellin responses, whereas the stress-responsive elements included low-temperature and drought responses. Moreover, some specific transcription factor-binding sites, such as W-box, MYB, and MYC, were identified in the promoters. Taken together, these results indicated that the seven CsGSTs could be induced by environmental and hormonal signals.

### Antioxidant enzyme activity analysis of cucumber seedlings under cold stress

**FIGURE 7:**
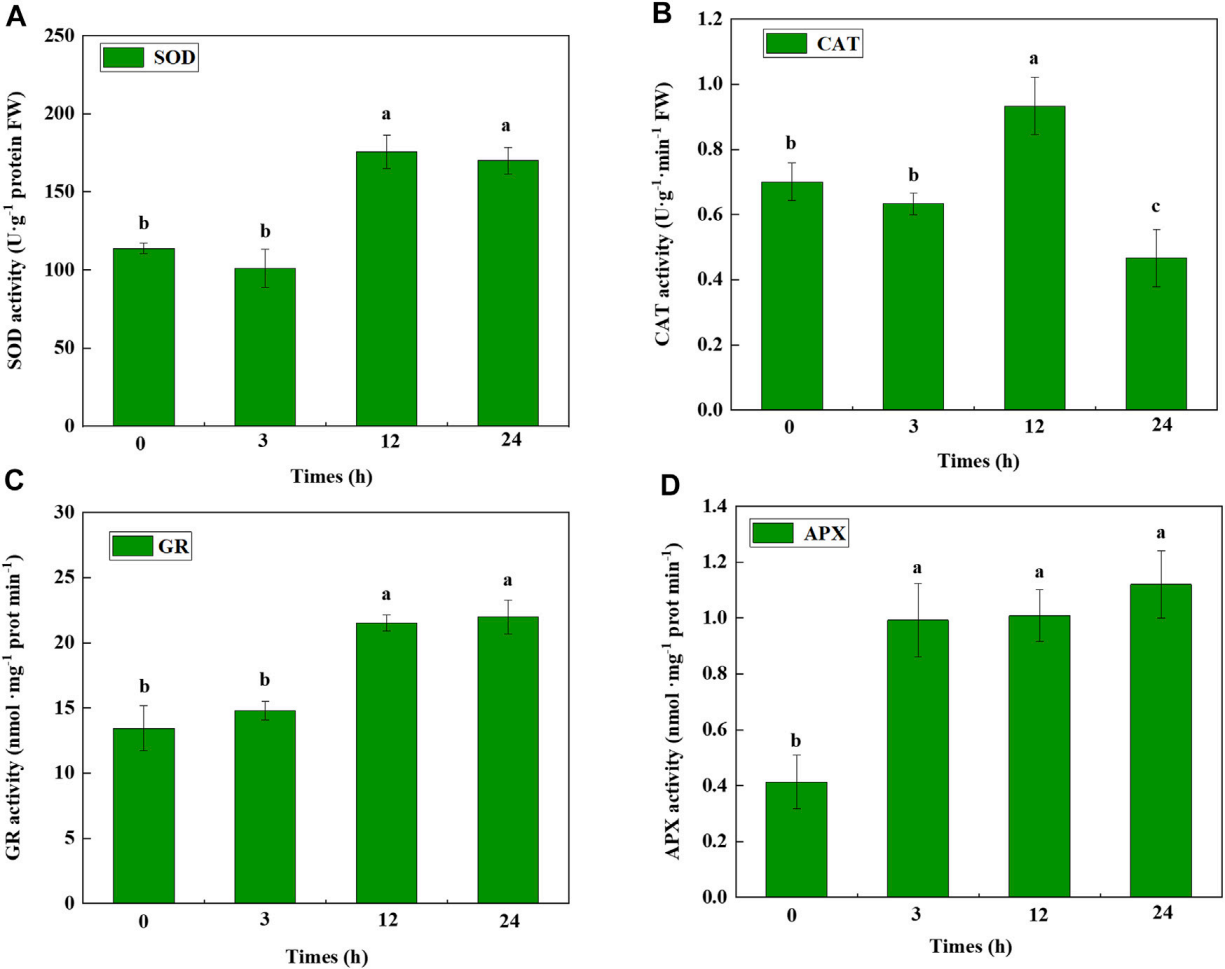
Antioxidant enzyme activity of cucumber seedlings under cold stress. **(A)** SOD, **(B)** CAT, **(C)** GR, and **(D)** APX activity determined at different hours after cold stress. Data were analyzed from three independent biological replicates and represent the mean ± SE. Different letters indicate significant differences at *p* < 0.05. SOD, superoxide dismutase; CAT, catalase; GR, glutathione reductase; APX, ascorbate peroxidase.

We evaluated four antioxidant enzymes in cucumber seedlings under cold-stress conditions (Figure 7). The results showed that SOD and GR activities were significantly increased 12 h after cold treatment. APX activity sharply improved 3 h after cold treatment. CAT activity significantly increased 12 h after cold treatment and decreased 24 h after cold treatment. These results indicated that when cucumber seedlings are subjected to cold stress, antioxidant enzymes are induced to protect the seedlings from serious damage.

## Discussion

GSTs belong to a large gene family ubiquitously present in plants and have been identified in diverse species (Supplementary Table S5). There is a considerable difference in the number of GST proteins in different species. However, the number of GSTs in cucumber (46), *Cucumis melo* (49), and *Cucurbita maxima* (32) are comparable, implying that the evolution of GSTs in Cucurbitaceae is conserved. In the present study, we identified 46 members of the GST gene family in the cucumber genome, which were divided into 11 classes. Phylogenetic analysis showed that the members share high similarity among the same classes of GSTs in different species, indicating these gene classes have shared ancestry (Figure 3). Gene family expansion in plants is caused by tandem, segmental, and genome duplications (Kong et al., 2007). Among the identified classes, the tau class was the biggest subclass with 22 members, possibly due to tandem gene duplication on chromosomes 3 and 4. In contrast, the MGST, TCHQD, GHR, DHAR, and GST2N classes contained fewer members, possibly due to the scattering of these classes on different chromosomes, prohibiting tandem duplication (Supplementary Figure S2).

With respect to gene structure, the CsGST genes in the same class were similar in the number and position of introns and exons and had similar motifs (Figure 1). Therefore, extensive functional redundancy may appear within the GST gene family. For example, the tau class of GSTs provides protection against salt stress in *A. thaliana* and soybean (Kissoudis et al., 2015; Xu et al., 2015). Likewise, the lambda class of GSTs contributes to the defense against salinity stress in soybean and rice (Kumar et al., 2013; Chan and Lam, 2014). The partial reason for differences in GST classes in different species may be the loss of functionally redundant genes.

The expression pattern of CsGSTs under cold stress was analyzed and divided into three distinct groups with different expression patterns (Figure 4A). The CsGST members in the second group were significantly upregulated during cold treatment; among them, 18 of the 21 members belonged to the tau subclass. Compared with those at 0 h, the differentially expressed CsGSTs at 3, 12, and 24 h after cold treatment were 13, 19, and 16 with the tau subclass accounting for 9, 13, and 11, respectively. Venn analysis indicated that seven CsGSTs were commonly differentially expressed during the entire period of cold stress, and five of the seven CsGSTs belonging to the tau subclass were significantly upregulated (Figure 5D). Taken together, we speculate that the tau subclass members play an important role in responding to cold stress. Similarly, seven tau subclass members in pumpkin (Kayum et al., 2018) and three in cabbage (Vijayakumar et al., 2016) are considered candidate genes involved in responding to cold stress.

For further functional analysis, we functionally annotated the differentially expressed CsGSTs under cold stress using GO annotation and KEGG pathway enrichment analysis. The results implied that the CsGSTs were enriched in “glutathione metabolism,” “arachidonic acid metabolism,” and the “tosine metabolism” pathway, and the “glutathione metabolism” pathway played a vital role in resisting cold stress. The results are consistent with a previous study that revealed the involvement of CmGSTs in the “glutathione metabolism” pathway during cold stress response in Hami melon (Song et al., 2021). Therefore, we speculated that CsGSTs could bind to and activate the thiol group of GSH, and the S-glutathionylated xenobiotics would be sequentially processed to participate in the cold stress response. Surprisingly, two differentially expressed CsGSTs (CsGHR1 and CsTCHQD1) were not enriched in any pathway; therefore, other mechanisms may participate in cold stress.

To explore the regulatory factors of candidate CsGSTs, their promoters were analyzed using PlantCARE and New PLACE online tools (Figure 6). The results showed that CsGST promoters roughly contained three types of regulatory *cis*-elements, namely light and phytohormone response elements and transcription factor-binding sites, implying that CsGSTs were induced by environmental and phytohormone signals and specific transcription factors. Previous studies have reported that some GST genes are regulated by these signals. For example, *PcGST1* induced by auxin and UV-B light is involved in the signal transduction pathway to *CHS* (Loyall et al., 2000). Furthermore, *AtGSTU17*, regulated by light and hormones, is involved in modifying seedling development in *A. thaliana* (Jiang et al., 2010), Similarly, *CsGSTU8* is regulated by CsWRKY48 and positively responds to drought stress (Zhang et al., 2021). *PpGST1* activated by PpMYB10.1 participates in anthocyanin accumulation in peach (Zhao et al., 2020). However, whether the seven CsGST candidates are regulated by light, hormones, and specific transcription factors and how their expression is regulated in cucumber requires more in-depth studies.

In the present study, antioxidant enzyme activities and CsGSTs were induced by cold stress. Nevertheless, the indirect and direct associations between CsGSTs and antioxidants require further study. A previous study showed that *MsGSTU8* overexpression in tobacco could enhance the activities of SOD, POD, and CAT, improving the saline-alkali tolerance of transgenic tobacco (Du et al., 2019). Similarly, *LeGSTU2* overexpression in tobacco improved SOD, POD, and GST activities, thereby enhancing plant resistance to salt and drought stresses (Xu et al., 2015). Moreover, *ThGSTZ1* overexpression in *A. thaliana* increased GST, SOD, and POD levels for scavenging ROS, improving the drought and salinity tolerance of transgenic *A. thaliana* plants (Yang et al., 2014). Therefore, we hypothesized that when cucumber seedlings are subjected to cold stress, phytohormones are induced that serve as signal molecules to stimulate the expression of CsGST genes. Besides their role as antioxidant enzymes involved in cold stress, CsGST genes could also cloud the effect of antioxidant enzyme activity on resisting cold stress *via* the increased expression of their transcripts. The specific transcription factors induced by cold stress could bind to CsGST candidate promoters to regulate their expression, thereby promoting cold stress tolerance. However, further studies are required to identify the regulation network of CsGSTs in response to cold stress.

## Conclusion

We performed a genome-wide analysis of the *CsGST* gene family in cucumber and explored their role in responding to cold stress. A total of 46 *CsGST* were identified, and their gene structure, chromosomal distribution, protein characteristics, and evolutionary relationship were analyzed in detail. In addition, seven candidate genes that may be involved in cold stress were selected based on the transcriptome analysis, and their promoters were analyzed to predict the factors that could regulate their expression. Finally, antioxidant enzymes were evaluated to explore their relationship with CsGST. However, the molecular mechanism underlying the response of *CsGST* to cold stress needs to be further verified through transgenic technology. Taken together, our results provide a solid foundation for the future investigation of *CsGST* in cucumber.

## Data Availability

The datasets presented in this study can be found in online repositories. The names of the repository/repositories and accession number(s) can be found below: GEO accession GSE210703.
