# Supplementary material for: Genome-wide identification and expression analysis of glutathione S-transferase gene family to reveal their role in cold stress response in cucumber
Source: Front Genet. 2022 Sep 29;13:1009883. doi: 10.3389/fgene.2022.1009883 (PMC9556972; doi:10.3389/fgene.2022.1009883)
Supplement: Supplementary file 3 [file Table5.DOCX]

Supplementary Material

## Supplementary Figures


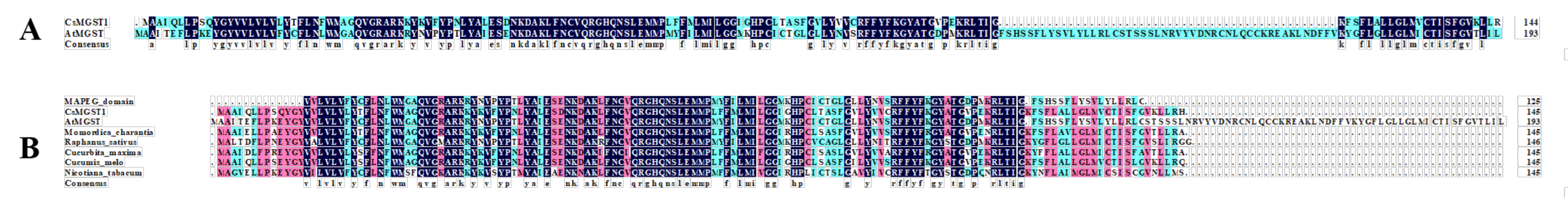


**Supplementary Figure 1.** Sequences alignment. (A) amino acid sequence alignment of CsMGST1 and AtMGST. (B) Multiple sequences of MAPEG domains were aligned among other species.


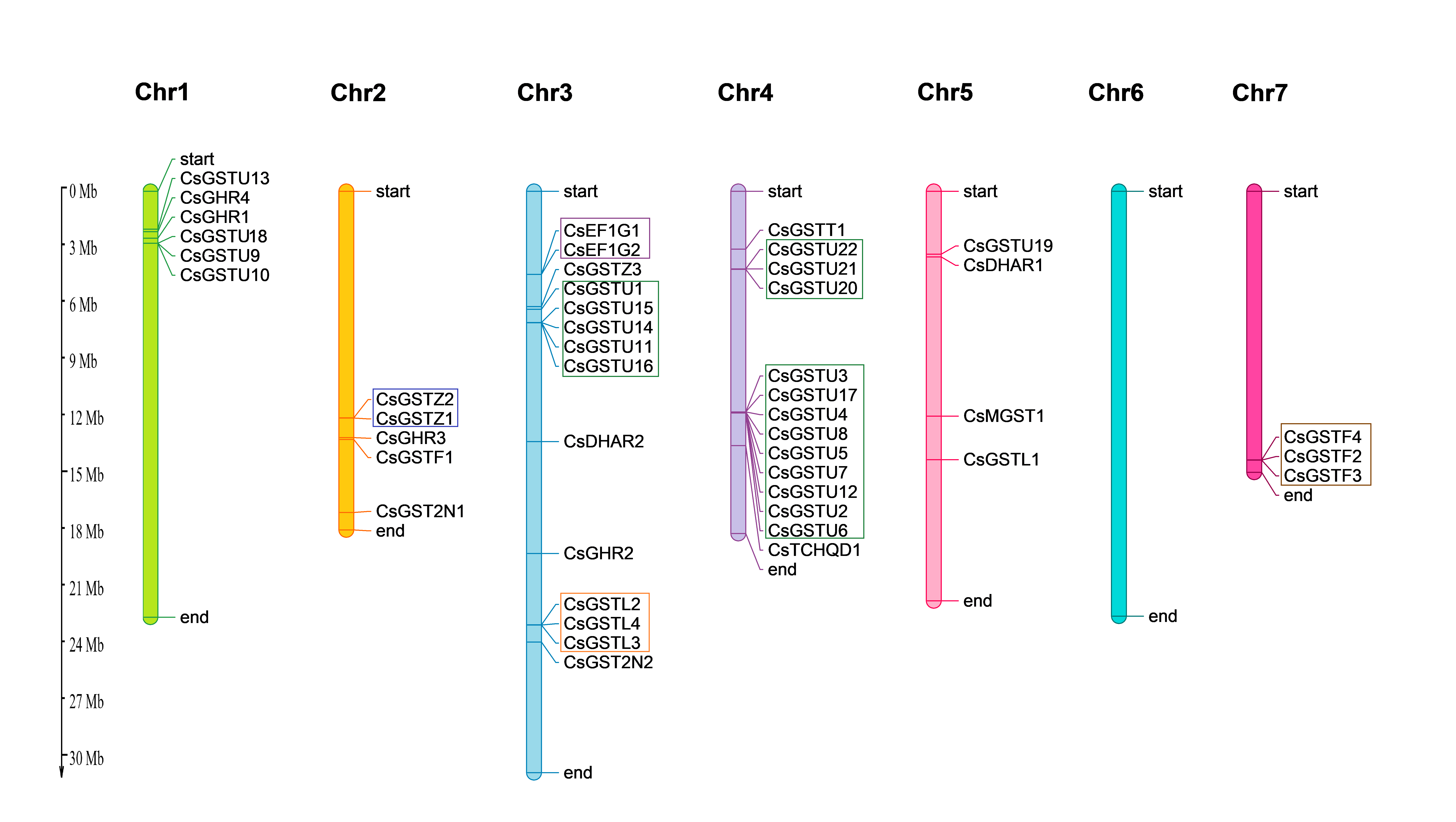


**Supplementary Figure 2.** Chromosomal distribution of GST genes in cucumber. The gene names are on the right side of the chromosomes. The seven CsGST clusters are indicated by the different colors. The scale on the left indicates the size of the chromosome.


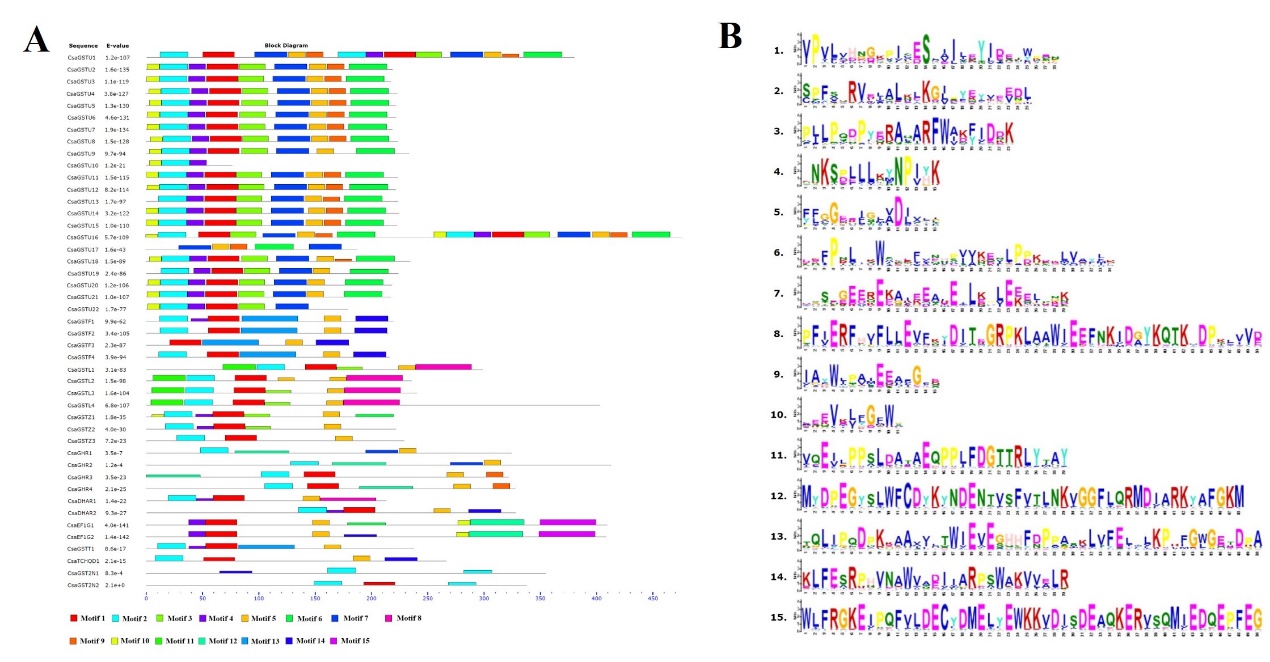


**Supplementary Figure 3.** The distribution of conserved motifs of GST proteins in cucumber. (A) The different conserved motifs of the 46 cucumber proteins are shown in different colors. The scale at the bottom of the figure indicates the position of the conserved motifs. (B) The amino acid composition of the 15 conserved motifs of GST.
