# Supplementary material for: Genome-wide identification and expression analysis of glutathione S-transferase gene family to reveal their role in cold stress response in cucumber
Source: Front Genet. 2022 Sep 29;13:1009883. doi: 10.3389/fgene.2022.1009883 (PMC9556972; doi:10.3389/fgene.2022.1009883)
Supplement: Supplementary file 6 [file Table6.DOCX]

Supplemental table 5 The GST members in different species

| Species | NO. of GST | Reference |
| --- | --- | --- |
| *Brassica oleracea* | 65 | Vijayakumar et al., 2016 |
| *Ipomoea* *batatas* L. Lam. | 42 | Ding et al., 2017 |
| *Pyrus bretschneideri* | 62 | Wang et al., 2018 |
| *Cucurbita maxima* | 32 | Kayum et al., 2018 |
| *Brassica napus* | 179 | Wei et al., 2019 |
| *Radish* sativus | 82 | Gao et al., 2020 |
| *Tribolium castaneum* | 41 | Song et al., 2020 |
| *Cicer arietinum* | 51 | Ghangal et al., 2020 |
| *Glycine max* L. Merr. | 126 | Hasan et al., 2020 |
| *Cucumis melo* | 49 | Wang et al., 2020 |
| *Malus domestica* | 38 | Zhao et al, 2021 |
| *Medicago truncatula* L | 92 | Hasan et al., 2021 |
| *Prunus avium* L. | 67 | Sabir et al., 2022 |
| *Teinturier Grape* | 92 | Li et al., 2022 |
